# Satisfaction of Search Can Be Ameliorated by Perceptual Learning: A Proof-of-Principle Study

**DOI:** 10.3390/vision6030049

**Published:** 2022-08-10

**Authors:** Erin Park, Fallon Branch, Jay Hegdé

**Affiliations:** 1 Department of Psychological Sciences, College of Science and Mathematics, Augusta University, Augusta, GA 30912, USA; 2 Department of Neuroscience and Regenerative Medicine, Medical College of Georgia, Augusta University, Augusta, GA 30912, USA; 3 Department of Ophthalmology, Medical College of Georgia, Augusta University, Augusta, GA 30912, USA; 4 The Graduate School, Augusta University, Augusta, GA 30912, USA; 5 James and Jean Culver Vision Discovery Institute, Augusta University, Augusta, GA 30912, USA

**Keywords:** airport baggage screening, camouflage-breaking, deep learning, game hunting, military reconnaissance, pathology, pattern recognition expertise, radiology, segmentation, subsequent search miss (SSM), visual search

## Abstract

When searching a visual image that contains multiple target objects of interest, human subjects often show a satisfaction of search (SOS) effect, whereby if the subjects find one target, they are less likely to find additional targets in the image. Reducing SOS or, equivalently, subsequent search miss (SSM), is of great significance in many real-world situations where it is of paramount importance to find all targets in a given image, not just one. However, studies have shown that even highly trained and experienced subjects, such as expert radiologists, are subject to SOS. Here, using the detection of camouflaged objects (or camouflage-breaking) as an illustrative case, we demonstrate that when naïve subjects are trained to detect camouflaged objects more effectively, it has the side effect of reducing subjects’ SOS. We tested subjects in the SOS task before and after they were trained in camouflage-breaking. During SOS testing, subjects viewed naturalistic scenes that contained zero, one, or two targets, depending on the image. As expected, before camouflage-training, subjects showed a strong SOS effect, whereby if they had found a target with relatively high visual saliency in a given image, they were less likely to have also found a lower-saliency target when one existed in the image. Subjects were then trained in the camouflage-breaking task to criterion using non-SOS images, i.e., camouflage images that contained zero or one target. Surprisingly, the trained subjects no longer showed significant levels of SOS. This reduction was specific to the particular background texture in which the subjects received camouflage training; subjects continued to show significant SOS when tested using a different background texture in which they did not receive camouflage training. A separate experiment showed that the reduction in SOS was not attributable to non-specific exposure or practice effects. Together, our results demonstrate that perceptual expertise can, in principle, reduce SOS, even when the perceptual training does not specifically target SOS reduction.

## 1. Introduction

Satisfaction of search (SOS) refers to a visual search phenomenon in which if a viewer searching a visual scene finds one target (i.e., an object of interest), they are more likely to miss additional targets, if any, in the scene [1,2,3,4]. SOS is sometimes also referred to as subsequent search miss, or SSM [5,6].

SOS was originally reported in the context of a radiological examination of chest X-rays by practicing radiologists [1,2]. More recently, Fleck and colleagues have demonstrated that SOS is not idiosyncratic to the radiological examination of images, in that it can occur during a conventional visual search by non-professional subjects with no prior expertise in visual search [4].

SOS is a phenomenon of great interest not just from a purely scientific point of view, but also from a practical one. This is because SOS can lead to adverse outcomes in many real-world situations. For instance, if a radiologist finds one cancerous tumor in a chest X-ray and misses others, the patient will still face mortal danger. If, in a combat situation, a reconnoiterer in charge of finding camouflaged land mines in the path of an advancing infantry finds one land mine and misses others, it is not only practically useless, but also positively deadly, because any one of the unfound land mines is potentially just as dangerous as the one found.

For similar reasons, SOS can be a matter of life or death in many other real-world situations where a visual search for targets plays a key part, such as pathology, airport baggage screening, and game hunting. Thus, reducing or eliminating SOS has substantial real-life significance. However, no effective methods for reducing, much less eliminating, SOS actually exist, although previous studies have suggested some potentially useful conceptual approaches to it [7].

The present preliminary study explores a novel approach to reducing SOS, namely perceptual learning. Briefly, perceptual learning refers to “long-lasting changes in perception that result from practice or experience” [8] (for reviews, see, [9,10,11,12]). There is a broad consensus on two properties of perceptual learning that are particularly relevant to the present study. First, by the aforementioned functional criterion that the changes be “long-lasting”, transient effects such as dark adaption of the eyes do not count as perceptual learning, but perceptual changes that occur in less than an hour [13], or within 100–200 trials, do count. Second, feedback is helpful, but not strictly necessary for perceptual learning [14]. That is, perceptual learning can occur without feedback (i.e., in ‘unsupervised learning’ settings) [10]. Indeed, mere exposure to visual stimuli can lead to perceptual learning [14]. It has been known since the landmark work of Gibson and Gibson in the 1950s that perceptual learning can occur without feedback [15]; practice and exposure without supervision (in the machine learning sense) can also result in perceptual learning [10,16].

The present study was motivated by previous reports that a search target that is initially hard to find becomes significantly easier to find with practice (see, e.g., [17,18,19] and the references therein). For instance, finding an intact circle among intersected circles, where each circle is intersected by a vertical line segment (i.e., finding an O-shaped target among Q-shaped distractors) tends to be difficult for naïve observers, so their reaction time increases with the number of distractors. However, with extended practice, subjects tend to find the target quickly regardless of the number of distractors. Thus, in this case, perceptual learning reduces the hindering effect of additional similarly shaped objects (in this case, the distractors), so that the target, in effect, ‘pops out’ [17,18,19].

We reasoned that perceptual learning could have a comparable effect in SOS where the targets all popout, so that finding multiple targets should be approximately as easy as finding a single target. This preliminary study was designed to test this hypothesis, namely that perceptual learning can reduce SOS. In this brief report, we test this hypothesis using camouflage-breaking as an illustrative example and show that perceptual learning does indeed reduce SOS in camouflage-breaking.

## 2. Experiment 1: Characterization of the Relationship between Perceptual Learning and SOS

Finding a target camouflaged against its background, or ‘camouflage-breaking’, involves perceptually distinguishing the target from its background [20,21,22,23,24,25]. As a first approximation, the task difficulty in camouflage-breaking arises from having to perceptually distinguish, or ‘segment’, the target from its background [22,23,25]. If a target is sufficiently well-camouflaged, it can be all but impossible to find it even if it is in ‘plain sight’ [22,25]. Thus, searching for a camouflaged object is a special case of a visual search [25,26].

By contrast, the task difficulty in conventional visual search stimuli, such as the aforementioned array with O- and Q-shapes, arises from having to distinguish the targets from the distractors and vice versa, and not from the difficulty of segmenting them. This is because both the target and the distractors are typically highly distinguishable from the background and from each other in conventional visual search stimuli [26].

Therefore, it does not necessarily follow from the aforementioned studies of perceptual learning that the effect of learning on conventional visual search generalizes to camouflage-breaking. Moreover, it is far from clear that the learning-dependent reduction in reaction times necessarily translates to a reduction in SOS. Thus, the aforementioned hypothesis is far from trivial, and it is important to empirically test it.

We have previously reported that naïve subjects with no prior training in, or aptitude for, camouflage-breaking can be trained to become expert camouflage-breakers using a ‘deep-learning’ paradigm [25]. Briefly, in this perceptual learning paradigm, subjects learn the statistical properties of the background (i.e., what the background ‘looks like’) using a large number of camouflage scenes (see ref. [25] for details; also see Section 2.1). Prior to the training, the subjects’ camouflage-breaking performance tends to be at chance levels, as expected. After the training, the subjects break camouflage at statistically significant levels.

**Figure 1:**
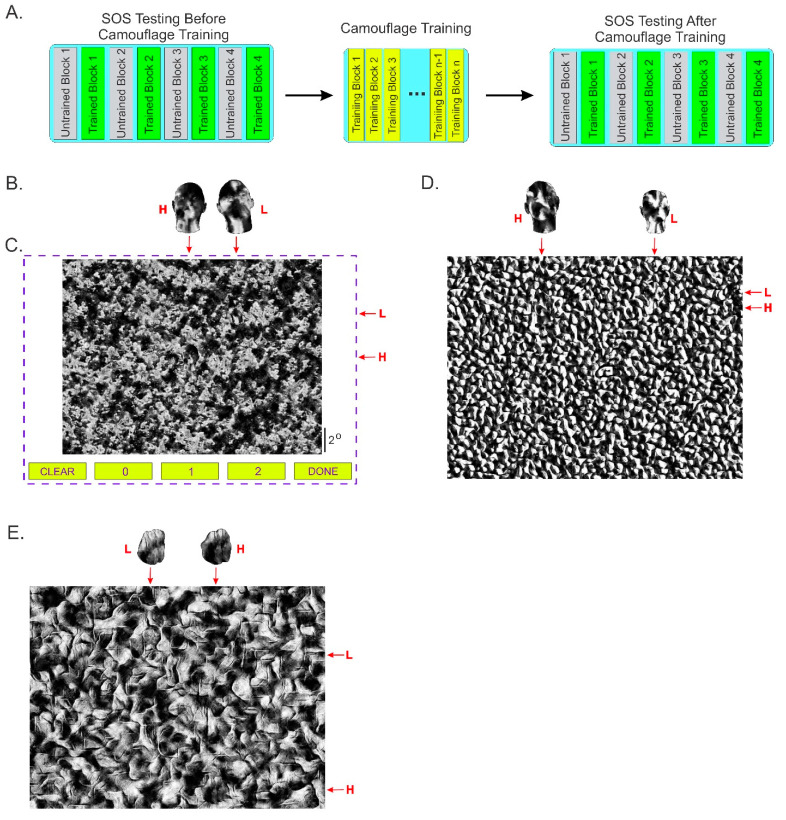
Study design. (**A**) Workflow of Experiment 1. At the start of the experiment, each subject’s baseline SOS was measured for both the designated trained and untrained textures (*far left*). Each subject then underwent as many blocks of camouflage training as necessary for them to reach criterion-level camouflage-breaking performance (*center*). The SOS testing was then repeated (*far right*). The order of the trained vs. untrained blocks was counter-rotated across subjects (not shown). (**B**) Exemplar high-saliency (H) and low-saliency (L) human head targets. For clarity, the targets are shown slightly more magnified than their actual size in the camouflage image. (*left* and *right*, respectively). (**C**) Exemplar SOS test screen with a camouflage image of the texture-type ‘foliage’. The image was of ‘L-and-H’ type in that it contained, in camouflaged form, one H target and one L target that are shown in isolation in panel B. To help the reader detect the targets, the actual horizontal and vertical locations of the targets are denoted by the corresponding *red arrows* outside the actual screen area (*dashed blue rectangle*). Needless to say, these cues were not available to the subject during the actual experiment. (**D**,**E**) Additional exemplar images of the L-and-H type, with head targets camouflaged against ‘nuts’ background (*panel* **D**) and digital embryo targets camouflaged against ‘fruit’ texture (*panel* **E**). Note that, of the three texture types shown in this figure, only the ‘foliage’ and the ‘fruit’ textures (panels **C**,**E**) were used in Experiment 1. The ‘nuts’ texture shown in panel (**D**) was not used in Experiment 1 (but was used in Experiment 2 below) but is shown in this figure for the sake of comparison. See text for additional details.

In this experiment, we take advantage of two specific properties of this perceptual training phenomenon that we have previously characterized: First, subjects can learn to break the camouflage of a target by learning the statistical properties of the background [27,28]. That is, once the subjects learn what the background ‘looks like’, they can tell if there is an object in a given scene that does not ‘belong’ in the scene. A second, interrelated property is that the improvement in camouflage-breaking performance transfers very well across the *targets*, but poorly across *backgrounds* [27,29]. Thus, when the subjects are trained to a criterion using images in which both the target and background had a given type of texture (e.g., ‘foliage’, see Figure 1) and their camouflage-breaking performance is tested using the same background texture (i.e., ‘trained texture’), their performance remains high. However, when the subjects are concurrently tested using a sufficiently different texture (e.g., ‘fruit’ or ‘nuts’, see Figure 1) in which they were not trained, the performance in these ‘untrained textures’ remains at baseline levels.

This experiment uses a simple variation of this train–test design to test the above hypothesis: We use one given texture to train the subjects in *camouflage-breaking* and test the subjects’ *SOS* before and after the training using both trained and untrained textures. If camouflage training does reduce SOS, then the post-testing SOS levels should be lower compared to the pre-training levels for the trained texture, but not for the untrained texture. We show that this is indeed the case.

### 2.1. Methods

#### 2.1.1. Subjects

A total of 15 subjects participated in this experiment. All were adult volunteers between 18 and 65 years of age with normal or corrected-to-normal vision. All persons who met the above criteria were enrolled; no other inclusion or exclusion criteria were used. All subjects gave written informed consent prior to participating in the study. All procedures related to study subjects were approved in advance by the Institutional Review Board (IRB) of Augusta University, where this study was carried out.

#### 2.1.2. Stimuli

We have previously described, in detail, the general procedures for generating and presenting camouflage stimuli used in this experiment [25]. The stimuli used in this experiment were created using the same basic approach (with slight modifications depending on whether the stimuli were created for camouflage training or SOS testing) as described below.

*Stimuli used during camouflage training.* These stimuli were created as described previously by us in detail (see ref. [27], especially Figure 1 therein). Briefly, the textures used in this study were synthesized using actual grayscale pictures of two different types of natural textures (tree foliage and fruit at a fruit stand) as input to a texture synthesizer developed by Portilla and Simoncelli [30,31]. We synthesized thousands of different bitmaps (or ‘instances’) for each of the aforementioned three texture types.

To create an individual camouflaged image with a target, we texture-mapped a target object using one instance of the texture type ‘foliage’, and digitally overlaid it at a pseudo-random location on a different instance of the same texture type. That is, the target (when present) always had the same texture as the background, which is essentially what helped camouflage it. The target, when present, had an equal probability of being either a human head (generated using the FaceGen toolkit; Singular Inversions, Toronto, Canada; facegen.com) or a type of novel 3D virtual object called a ‘digital embryo’ [32], depending on the image (see Figure 1 of [27]). To help further reduce target predictability, we randomly varied its spatial location (anywhere in the image as long as it was not clipped by the edge of the image), size (mean angular size, 0.5° ± 0.05° [SD]), and orientation (range of rotations the *x* and *z* axes, −45° to +45°; about *y*-axis, −90° to +90°). We have shown previously that these procedures together can produce camouflaged images in which the target is highly effectively camouflaged ‘in plain view’ against the background without shadows or occlusion [27,32].

We picked an equal-sized but non-overlapping subset of instances of the same texture type (i.e., foliage) to serve as stimuli that did not contain the target. Thus, the stimulus subsets that did vs. did not contain a target had the same background statistics, and contained, with equal probability, a single target or no target, depending on the image. When a target was present, it had a 50% chance of being a human head or a digital embryo.

We similarly created a large number of images using the ‘fruit’ texture. To help minimize potential confounds related to a particular texture type, we counterrotated the textures across subjects, so that each given subject was trained and tested using either of the above texture types (foliage, seven subjects; fruit, six subjects); no subject encountered both the texture types.

For either of the texture types, no single bitmap was used twice at any point during stimulus construction or during the trials, so that the subjects could not perform the task by any pixel-wise comparison of bitmaps, including remembering the bitmaps. The only way they could learn to effectively break the camouflage of the target was to learn the statistical properties of the background, i.e., what the given background type (say, foliage) ‘looked like’.

*Stimuli used during SOS testing*. These stimuli were created exactly as described above, except as noted otherwise. Briefly, each stimulus consisted of zero, one, or two targets (depending on the stimulus) composited against the background texture (see, e.g., Figure 1 for an example of the ‘fruit’ texture type). When a stimulus contained two targets, they were always of the same type (i.e., both were heads or both were digital embryos, but never one head and one digital embryo). The target/s, when present, had a 50% chance of being human head/s or digital embryo/s. The target/s (when present) had the same texture as the background (see [25]).

A given target, when present, was either a high-saliency target (‘H’) so that it was comparatively easy to find or a low-saliency (‘L’) target so that it was relatively difficult to find. The H and L targets were created solely by varying the density of their textures during the aforementioned texture mapping process (see, e.g., Figure 1A). Using pilot experiments, we adjusted the texture density of the H target so that, in the absence of the L target, naïve subjects detected the H target correctly (i.e., with correct localization) in approximately 75% of the trials on average. Similarly, we adjusted the texture density of the L target so that, in the absence of the H target, subjects detected it correctly (with correct localization) in approximately 30% of the trials. After this adjustment, the L targets were slightly more salient, and the H targets were far more salient, on average, than the targets in the aforementioned camouflage training stimuli.

#### 2.1.3. Task Paradigms

Depending on the trial block, the subjects performed one of two tasks: (i) Camouflage-breaking task, which the subjects performed during camouflage training/testing blocks, or (ii) SOS task, which the subjects performed during SOS testing blocks.

Before the subjects received any camouflage training, we tested their SOS and camouflage-breaking performance to serve as the baseline measurements (Figure 1A, *left*). After this, subjects received camouflage training (Figure 1A, *center*). After the subjects were trained to the criterion, the subjects’ SOS was tested exactly as before (1A, *right*).

#### 2.1.4. Camouflage Training Blocks

During these blocks (which consisted of 40 trials each), the subjects performed a camouflage-breaking task, the purpose of which was to train naïve, non-professional subjects to become expert camouflage-breakers. This task design was identical to that used in our earlier deep-learning study [25] except as noted otherwise. Briefly, during each trial, subjects were presented a single camouflage scene, which had a 50% chance of containing (or not containing) a single target (not shown). The target, when present, had a 50% chance of being a head or a digital embryo. Subjects were allowed unlimited time to view the stimulus and indicate, using a keypress, whether or not the stimulus contained a target. Following their response, subjects received auditory feedback, and were allowed unlimited time to re-scrutinize the stimulus in light of the feedback, after which they pressed a designated button to start the next trial. Subjects were not told what to learn or how to learn it, and they did not have to localize the target.

Each subject underwent as many training blocks as needed to achieve a criterion-level performance. The subjects were considered trained to a criterion if their discriminability (or *d’*) was ≥1.9 during at least two successive blocks on the same day. Discriminability is a standard signal theoretic metric that measures, in the present context, the extent to which the subjects can reliably distinguish the presence vs. absence of the target [33].

#### 2.1.5. SOS Testing Blocks

During these blocks (which consisted of 60 trials each; see Table 1), subjects performed an SOS task, which was a modified version of the task used by Fleck and colleagues [4]. Briefly, each trial began with the presentation of a small (0.3°), central fixation spot on a neutral gray background. When the subject established fixation and pressed a designated key to indicate trial readiness, the camouflage stimulus was presented along with on-screen response buttons at the bottom (Figure 1C). The subject was allowed ad libitum opportunity to view the image and indicate the location of the target/s, if found, using a mouse click. The location/s of the mouse click/s were tagged on the stimulus by small ‘X’ mark/s. If they so desired, subjects were able to clear all tags using the corresponding on-screen button (Figure 1C, *bottom left*). The subject also had to use the corresponding on-screen button to indicate the number of targets found in the image (Figure 1C, *bottom*). The subject finalized the response and ended the trial by pressing the ‘Done’ button (Figure 1C, *bottom right*).

Experiments were controlled and the data were collected using custom-written scripts in the Presentation scripting language (neurobs.com). Trials were presented in pseudo-random order within each trial block throughout the experiment. The onset of individual trials, trial blocks, and experimental sessions was self-paced by the subject throughout the experiment for maximum comfort. Prior to the actual data collection, subjects were shown exemplar camouflage training stimuli as well as exemplar SOS testing stimuli, and were shown several exemplary textured targets (i.e., human heads and embryos) in isolation. Subjects were informed that the exemplar images and targets were meant only to provide the subjects a general idea of what they looked like, and the actual images and target/s will differ from one trial to the next and from the exemplars shown. To help minimize stimulus predictability, subjects were not provided any information about the relative prevalence of any of the stimulus parameters. Subjects were able to perform practice trials ad libitum to familiarize themselves with the task paradigm. The data from the practice trials were discarded [25,27].

### 2.2. Data Analysis

We analyzed the data using custom-written scripts in the R language [34]. To analyze the camouflage-breaking performance during camouflage training blocks, we calculated a *d’* value for each successive training block of the given subject, which resulted in a learning curve [35] of *d’* values of length *n* for each subject, where *n* is the number of blocks needed for the given subject to reach criterion-level performance.

The analysis of SOS was carried out as described by Fleck et al. [4]. To measure the SOS prior to camouflage training, we pooled the data from all pre-training SOS blocks across all subjects. This resulted in a pool of 60 blocks of trials, each of which had 10 L-only trials and 10 L-and-H trials. Note that the fact that the two types of trials had the same number of trials (i.e., 10) in each block makes the analysis of the SOS data using raw trial counts straightforward, as we do below for the sake of simplicity.

For each block, we calculated the number of L-only trials during which the target was detected and correctly localized. We similarly calculated for each block the number of L-and-H dual target trials during which the L target was detected given that the H target was also detected (with correct localization of both targets). In other words, L-and-H trials in which both targets were found and correctly localized were counted as correct L-and-H trials, regardless of which target was localized first. This resulted in 60 trial counts each for L-only trials and for L-and-H trials. To measure pre-training SOS, we compared the two sets of 60 values each using a paired, 2-tailed *t*-test and determined the effect size using Cohen’s *d*. We similarly measured the SOS for the post-training blocks.

To determine the relative contributions of various experimental parameters to SOS, we compared the response counts before vs. after camouflage training using general linear modeling with generalized Hermite distribution (*glm.hermite* function, R library *Hermite*) [36].

#### A Priori and Post Hoc Power Analyses

Before initiating the present study, we carried out an *a priori* power analysis to estimate the requisite sample size for the comparison of primary interest, namely the number of correct responses during L-only trials vs. L-and-H trials before the camouflage training. This comparison is of primary interest, because it speaks to whether there was an SOS effect to begin with prior to the camouflage training.

To perform the above power analysis, we used the Cohen’s *d* effect size of 0.67 that we had empirically observed in a pilot study with the same study design as the expected effect size of SOS. This analysis indicated that a total of 31 paired data points each for L-only and for L-and-H conditions (pooled across all subjects and blocks) would be needed to achieve a power of 0.95 at a significance level of 0.05 using a paired, 2-tailed *t*-test. Our sample sizes (*n* = 60) were nearly twice this minimum requirement in all cases. Indeed, *post hoc* power analyses using the actual data indicated that our data achieved a power of >0.95 for the aforementioned primary comparison of interest.

### 2.3. Results

In this experiment, we measured SOS using the standard method described previously by Fleck and colleagues [4]. This entails comparing how often the subjects detect the L target when it is the sole target in the image (L-only trials) vs. how often they detect the L target when an H target is also present in the image (L-and-H trials). To the extent the presence of an H target makes the detection of the L target more likely, i.e., if there is SOS, subjects would, on average, tend to correctly detect the L target in the L-and-H trials less often than in L-only trials. The conventional *t*-statistic is a useful numeric measure of this effect [4].

#### 2.3.1. Subjects Show Substantial SOS before Camouflage Training

**Figure 2:**
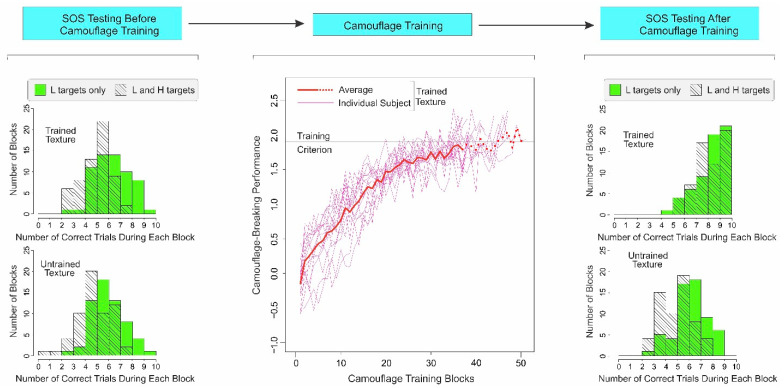
Results of Experiment 1. The temporal order of the three main phases of the experiment is schematically shown at the *top.* The results of SOS testing before and after camouflage training are shown in histogram form in the *far left* and *far right* columns, respectively. In either column, the *top* and *bottom histograms* show SOS data for the *trained* and *untrained textures*, respectively. Each histogram shows SOS as the comparison between the correct responses during the L-only trials (*green histogram*) vs. correct responses during the L-and-H trials (*hatched trials*). The extent to which the average response during the L-only trials exceed the responses during the L-and-H trials is a standard measure of SOS [4]. The *middle panel* of the figure shows the camouflage-breaking performance (as measured by *d’*) during successive blocks of camouflage training for individual subjects (*thin pink lines*; see *inset*) and the subject sample as a whole (*thick red line*). Note that some subjects reached criterion (*horizontal black line*) with fewer training blocks, so the effective sample size decreased during subsequent blocks, a fact denoted by the *thick dotted line.* See text for additional details.

The distributions of subjects’ responses to the two types of trials for the trained texture prior to camouflage training are shown in histogram form at the *top left* of Figure 2 (see legend for details). The subjects did indeed correctly detect the L target during the L-and-H trials (*M* = 5.43, *SD* = 1.28) significantly less well than during the L-only trials (*M* = 6.77, *SD* = 1.49; paired two-tailed *t*-test, *t*(59) = 4.91, *p* < 0.001). The size of this effect as measured by Cohen’s *d* statistic was 0.96 (95% confidence interval [CI] = 0.58 and 1.34), indicating that the SOS in this case was quite large.

We obtained a similarly large SOS effect for the untrained texture before the camouflage training (Figure 2, *bottom left*; L-and-H trials: *M* = 5.25; *SD* = 1.45; L-only trials: *M* = 6.42; *SD* = 1.41; paired two-tailed *t*-test, *t*(59) = 4.39, *p* < 0.001; Cohen’s *d* = 0.82; CI = 0.44 and 1.19). Together, these results demonstrate that camouflage images can produce a substantial SOS effect in untrained subjects.

In our previous studies on perceptual learning that used a two-alternative forced choice (2AFC) task paradigm, we reported that prior to camouflage training, a majority of the subject’s incorrect responses are attributable to false alarms (or false positives), where the subjects mistake image regions with high visual saliency for targets [27,28,29,37]. During successful camouflage training, the proportion of false alarms tends to decrease systematically, thus contributing to the improvement in performance as measured by *d’* [27,28,29,37].

The SOS task is much more complex, in that the number of possible response alternatives is quite large. However, we did observe a comparable effect during the SOS task prior to camouflage training. One of the most common categories of such errors is shown in Supplementary Figure S1, where the *green histogram* at the *top left* denotes those L-only trials for the trained texture in which the subjects reported the image had a single target but incorrectly identified its location (*M* = 2.55, *SD* = 1.14). The corresponding *hatched histogram* denotes those L-and-H trials in which the subjects reported the image had two targets but incorrectly identified both their locations (*M* = 2.52, *SD* = 0.74; two-tailed *t*-test, *t*(59), *p* = 0.86). As expected, the results for the untrained texture were similar (Supplementary Figure S1, *bottom left*; L-only trials: *M* = 2.80, *SD* = 1.12; L-and-H trials: *M* = 2.70, *SD* = 0.79; two-tailed *t*-test, *t*(59) = 0.574, *p* = 0.568).

#### 2.3.2. Camouflage-Breaking Performance Systematically Improves during Camouflage Training

During camouflage training, the subjects were trained to criterion in the camouflage-breaking task. Note that the subjects were not trained in the SOS task *per* se in that the task and the underlying camouflage images used during the camouflage training were substantially different from those used for SOS testing. Most notably, the images during this phase consisted of a single camouflaged target (see Section 2.1 for details). Pilot studies (not shown) indicated that the saliency of the targets in these images was even lower than that of L targets in the images used for SOS testing (for the underlying rationale, see Section 2.1). Subjects took an average of 42.67 ± 4.92 [SD] training blocks and an average of 10.87 ± 1.45 training days to reach criterion-level performances.

As expected from our previous studies [27,28,29], the camouflage-breaking performance, as measured by *d’*, monotonically increased over successive blocks of training (Figure 2; *middle panel*), accompanied by a concomitant decrease in false alarm rates (not shown). Note that such a systematic change in perceptual performance is another important criterion of perceptual learning [11,12].

#### 2.3.3. After Camouflage Training, SOS Is Reduced for Trained Texture, and Not for Untrained Texture

After camouflage training, the SOS for the trained texture was reduced to statistically insignificant levels (Figure 2, *top right*; L-and-H trials: *M* = 8.62; *SD* = 1.25; L-only trials: *M* = 8.73; *SD* = 1.31; paired two-tailed *t*-test, *t*(59) = 0.48, *p* > 0.634; Cohen’s *d* = 0.09, CI = −0.27 and 0.45). However, the SOS for the *untrained* texture remained high (Figure 2, *bottom right*; L-and-H trials: *M* = 5.40; *SD* = 1.36; L-only trials: *M* = 6.62; *SD* = 1.42; paired two-tailed *t*-test, *t*(59) = 5.12, *p* < 0.001; Cohen’s *d* = 0.88; CI = 0.50 and 1.26).

This selective reduction was not idiosyncratic to the particular textures used as trained vs. untrained textures for a given subject, because the underlying textures were counterrotated across subjects (see Section 2.1). Thus, the reduction in SOS was specific to the texture in which the subjects obtained camouflage training.

General linear modeling of the response counts confirmed the above result that the response counts changed across both the texture type (i.e., trained vs. untrained texture) and training status (before vs. after camouflage training (see Table S1 in Supplementary Materials).

### 2.4. Discussion

As expected, subjects showed a significant SOS effect before they were trained in camouflage-breaking. Thus, our experimental setup did succeed in reproducing the SOS effect, and the magnitude of this SOS effect was broadly comparable to that previously reported for conventional visual search by previous studies [4].

We also found that camouflage training greatly reduced the SOS effect, indicating that perceptual expertise reduces SOS. Importantly, the reduction in SOS was specific to the trained texture because the same subjects continued to show SOS when they were tested using the textures in which they were not trained.

Notably, the reduction in SOS occurred as an indirect side effect of camouflage training, in that subjects were not specifically trained to reduce their SOS. Indeed, neither the SOS task nor the SOS stimuli were used to train the subjects (see Section 2.1).

Collectively, these results confirm the above hypothesis that perceptual training can reduce SOS in camouflage-breaking.

## 3. Experiment 2: Reduction in SOS Is Not Attributable to Non-Specific Learning and Other Exposure Effects

The aforementioned results leave open the possibility that non-specific learning effects contributed, at least in part, to our results. For instance, familiarity with camouflaged scenes caused by repeated viewing of the images may have made it easier for subjects to find even low-saliency targets in general.

It is important to distinguish specific vs. non-specific exposure effects. In the context of our study, specific exposure effects are those that result from repeated exposure to the specific type of camouflage texture in which the subject was trained, with specific (i.e., reliable) feedback, to break camouflage. In principle, some of the improvement in camouflage-breaking performance during such training may arise simply due to the fact that the subject was repeatedly exposed to such images. Indeed, previous studies, including ours, have shown that such learning can occur even in the absence of feedback [10,14]. Previous studies have also shown that such learning is specific to the specific type of images to which subjects were exposed; such exposure does not result in learning for images that are sufficiently different from the original images.

On the other hand, non-specific exposure effects are those improvements in the camouflage-breaking performance that could have occurred by repeated exposure to any type of camouflaged images, or by carrying out a large number of trials (practice effects), etc.

The present experiment tested the hypothesis that the reduction in SOS was not attributable to non-specific exposure effects. To this end, we trained subjects in one texture (‘trained texture’) using feedback that accurately reflected whether the preceding image contained a camouflaged target or not. The same subjects were also concurrently trained in a different texture (‘untrained texture’) using *random* feedback. We examined the SOS for the two textures before and after the training, as detailed in Methods.

### 3.1. Methods

This experiment was identical to Experiment 1 above, except as noted otherwise. Each methodological variation from Experiment 1 was intended to help measure the contribution, if any, of a confounding variable.

#### 3.1.1. Subjects

A total of eight subjects (none of whom participated in Experiment 1) participated in this experiment.

#### 3.1.2. Stimuli

In this experiment, the stimuli used two different types of textures: ‘foliage’ (Figure 1C) and ‘fruit’ (Figure 1E). The retinal size of the stimuli in this experiment was 25% larger than that in Experiment 1; this was due to the fact that the viewing distance in this experiment was shorter by 25% in this experiment.

#### 3.1.3. SOS Testing Blocks

The experimental conditions differed from those in Experiment 1 in two respects. First, each subject performed eight blocks of trials each for the trained and untrained textures. Second, for either texture, the prevalence of the ‘No target’ condition was 25% in this experiment (as opposed to 16.67% in Experiment 1) as shown in Table 2.

In this experiment, the prevalence of the conditions was adjusted so that the subjects had the same chance of encountering any given condition. In contrast, in Experiment 1, the subjects had the same chance of encountering a trial without a target as encountering a trial with target/s (see Table 1). We varied the prevalence parameter across experiments in order to test whether the reduction in SOS, if any, shows any dependence on target prevalence [4,38].

Note that the numbers and the overall prevalence of the trials corresponding to the two conditions of primary interest, namely the L-only and the L-and-H conditions, remained the same as in Experiment 1.

#### 3.1.4. Camouflage Training Blocks

This experiment differed from Experiment 1 in three respects. First, each subject was trained in two different textures (as opposed to one in Experiment 1). For four of the eight subjects, fruit served as the trained texture and foliage served as the nominally untrained texture, as explained below. The trained and untrained textures were switched for the remaining four subjects. Since the data did not significantly differ between the two subsets of subjects (not shown), we pooled the results across all subjects.

Second, feedback was provided for both textures. For the trained textures, the feedback accurately reflected the presence or absence of a target. For the ‘untrained’ texture, the feedback was random, so it provided no reliable information as to whether or not the given stimulus contained a target.

It is important to note that our IRB has determined that this does not constitute deception under the applicable regulations and policies.

Third, the training blocks (40 trials each) for the two texture types were alternated, so that each block for the trained texture was preceded and succeeded by a block for the untrained texture, and *vice versa.* A given subject was considered trained to the criterion if they reached a *d’* ≥ 1.9 in two successive blocks of the same texture type on the same day for the trained texture, untrained texture, or both. As an empirical matter, however, each subject reached the criterion in the *trained* texture first, and training in both the trained and untrained texture types was stopped at that point. Note that these two facts together meant that the number of training blocks that a given subject underwent for one texture could be slightly different than the number of blocks for the other texture.

### 3.2. Results

**Figure 3:**
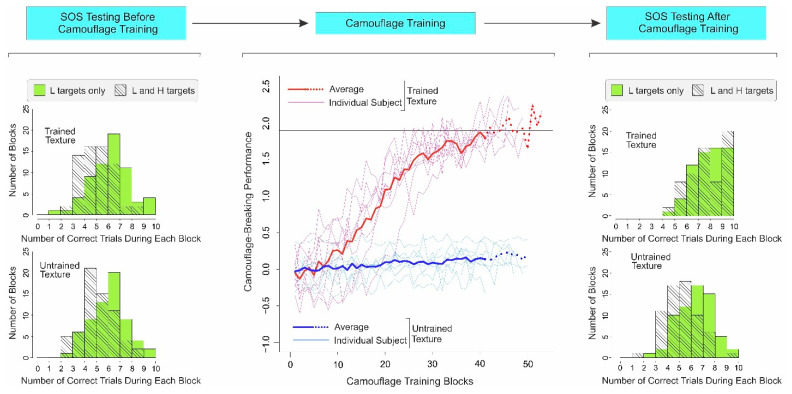
Results of Experiment 2. The results are plotted using the same conventions as in Figure 2. The only difference is that in this experiment, subjects were also trained in a second, nominally untrained texture using random feedback (*thin aqua lines* and *thick blue line*, *middle panel*). See text, for additional details.

As expected from Experiment 1 above, subjects showed substantial SOS for both trained and untrained textures before camouflage training in this experiment as well (Figure 3, *far left column*; Trained texture, L-and-H trials: *M* = 5.56; *SD* = 1.36; L-only trials: *M* = 6.66; *SD* = 1.67; paired two-tailed *t*-test, *t*(63) = 4.13, *p* < 0.001; Cohen’s *d* = 0.72; CI = 0.36 and 1.08; Untrained texture, L-and-H trials: *M* = 5.64; *SD* = 1.42; L-only trials: *M* = 6.53; *SD* = 1.53; paired two-tailed *t*-test, *t*(63) = 3.62, *p* < 0.001; Cohen’s *d* = 0.60; CI = 0.25 and 0.96).

As expected from Experiment 1, when subjects received accurate feedback (i.e., for the trained texture), camouflage-breaking performance systematically increased during camouflage training for each subject individually (Figure 3, *middle column*; *thin pink lines,* see *inset*) and across all subjects (*thick red line*). Subjects took an average of 45.88 ± 4.02 training blocks and an average of 12.0 ± 1.07 training days to reach the criterion.

#### Controlling for Exposure Effects of Camouflage Training

To examine the potentially confounding effects of specific and non-specific exposure, we concurrently trained the subjects in a different texture, for which they received random feedback, so that it provided no reliable information as to the presence or absence of the target in the stimulus (see Section 3.1). For this nominally untrained texture, the camouflage-breaking performance failed to increase (Figure 3, *middle column*; *thin aqua lines* and *thick blue line*), when the training was stopped because the subjects concurrently reached criterion-level performance for the trained texture (Figure 3, *middle column*; *thin pink* lines and *thick red line*). The fact that reliable feedback is needed for the subjects to successfully learn to break camouflage is consistent with our earlier reports [28,29,39].

Note that the subjects received the same overall level of exposure for the untrained texture as they did for the trained texture. Thus, improvements in camouflage-breaking performance for the trained texture are not attributable to exposure to a specific texture, or to non-specific effects stemming from repetition or familiarity with the task, etc. This is also consistent with our earlier results that showed that the effects of camouflage training transfer poorly, i.e., they are specific to the trained texture [28,29,39].

After camouflage training, the subjects’ SOS was greatly reduced for the trained texture (Figure 3, *top right*; L-and-H trials: *M* = 8.25; *SD* = 1.51; L-only trials: *M* = 8.39; *SD* = 1.30; paired two-tailed *t*-test, *t*(63) = 0.58, *p* > 0.565; Cohen’s *d* = 0.10; CI = −0.25 and 0.45), consistent with the results of Experiment 1 above. However, the SOS remained high for the nominally untrained texture, i.e., the texture in which the subjects received random feedback (Figure 3, *bottom right*; L-and-H trials: *M* = 5.73; *SD* = 1.39; L-only trials: *M* = 6.83; *SD* = 1.48; paired two-tailed *t*-test, *t*(63) = 4.38, *p* < 0.001; Cohen’s *d* = 0.76; CI = 0.40 and 1.12). The distributions of the aforementioned category of errors are shown in Supplementary Figure S2.

General linear modeling of the response counts in this experiment also confirmed for this experiment (as it did for Experiment 1) that response counts changed across both texture type (i.e., trained vs. untrained texture) and training status (before vs. after camouflage training) (see Table S2 in Supplementary Materials).

### 3.3. Discussion

The results of this experiment demonstrate that the reduction in SOS does not occur when the subjects underwent the same overall level of practice and repetition or when they received the same overall level of training and the same overall amount of feedback. Thus, the reduction in SOS was specific to the texture in which the subjects’ perceptual performance actually improved.

## 4. General Discussion

### 4.1. Perceptual Learning Reduces SOS

Our results demonstrate, for the first time, that perceptual training can reduce SOS. On the one hand, the subjects showed significant levels of SOS before camouflage training. After the subjects were trained to the criterion with accurate feedback, the subjects’ SOS was statistically indistinguishable from zero. On the other hand, the reduction in SOS did not occur when the subjects were not trained at all in a given texture (as in Experiment 1), or when they were ‘sham-trained’ with unreliable feedback (as in Experiment 2). Taken together, this evidence shows that the reduction in SOS is attributable to a training-dependent increase in camouflage-breaking performance, and not to non-specific effects such as the training itself or non-specific practice effects.

Two additional aspects of our results are worth noting. First, our results constitute the first demonstration of SOS in the specific context of camouflage. That is, they confirm and extend the previous reports of SOS in other contexts [1,2,3,4] to camouflage-breaking. Second, the reduction in SOS in our case was essentially a side effect of camouflage training, in that the training did not involve, and was not designed to *per se* reduce, SOS. That is, no special training was needed to reduce SOS; the reduction of SOS occurred as a matter of course during camouflage training.

Needless to say, all the above findings are of considerable practical significance in those real-life contexts where camouflage-breaking is of paramount importance, such as combat situations and game hunting.

This preliminary study was not designed to address the mechanisms by which improving camouflage-breaking performance results in a reduction of SOS. However, two observations about potential mechanisms are worth noting. First, the reduction in SOS—that is, improved detection of the L target in L-and-H stimuli—may be brought about by changes in the subject’s sensitivity, criterion, or both. Many previous studies have reported this effect in perceptual learning in general [40,41,42,43]. We have previously found similar effects in camouflage learning using L-only stimuli [28,29]. Second, expert camouflage-breakers report the subjective perception that the camouflage targets perceptually stand out, or ‘pop-out’, from their background, making them highly salient, i.e., very easy to recognize. If this is true, it would have the effect of greatly reducing the difficulty of recognizing additional targets, because recognizing two high-saliency targets is arguably approximately as easy as recognizing just one high-saliency target. This is consistent with, although by itself does not prove, the view that attributes SOS to the cost of searching for targets in visual images [7,44].

Our experimental controls collectively demonstrate that non-specific exposure effects are unlikely to have played a significant role in the observed reduction in SOS, because the reduction was specific to the trained texture. However, it remains possible that *specific* exposure effects, such as unsupervised learning due to repeated exposure to, and practice with, the specific training stimuli may have contributed to the improvement in camouflage-breaking performance during camouflage training, in addition to the feedback-dependent (i.e., supervised) learning. As noted above (Section 1 and Section 3), both are *bona fide* perceptual learning effects. Our preliminary study was not designed to measure the relative contributions of the two types of learning to SOS reduction.

### 4.2. How Generalizable Is Learning-Dependent SOS Reduction?

Our results show that the levels of SOS were statistically indistinguishable from zero after camouflage training. However, it is important to note that our results do not imply, much less prove, that SOS can be eliminated altogether by perceptual learning.

Moreover, it is not clear whether or to what extent SOS in other types of visual search can be reduced by perceptual learning. For practical reasons, the present study did not address this important but forbiddingly broad question.

For instance, it is conceivable that the SOS reduction effect does not generalize to medical image perception, since SOS is known to occur in expert radiologists. Indeed, as noted above, the SOS effect was first reported in the context of radiology [1,2].

There are multiple, plausible, mutually non-exclusive explanations for why pattern recognition expertise does not reduce SOS in radiology. First, it is possible that perceptual expertise does reduce SOS in radiology, and the relatively small SOS levels observed in expert radiologists reflect ‘leftover SOS’. That is, the SOS levels reported in practicing radiologists reflect the residual SOS effect that remains after the extensive clinical training in pattern recognition that radiologists receive reduces their SOS to the maximum extent possible. The reason this reduction has not been documented before is plausibly that the changes in the magnitude of SOS, if any, as a function of clinical training in recognizing diagnostic image patterns have not been systematically studied before.

Second, it is conceivable that the SOS reduction does not occur in this case, because radiologists are not systematically trained in the background statistics of the medical images they search in the same way as camouflage-breakers were trained in our study. That is, it is possible that SOS reduction is specific to our training method.

A third, related possibility is that the background textures in medical images are somehow substantially different from the background textures in our images. For one thing, it is self-evidently true that the complexity of medical images is much higher compared to the complexity of our images. For one thing, medical images have complex global information (or, in machine learning terms, ‘image grammar’ [45,46]) that our camouflage images lack.

Finally, it is possible that the SOS reduction is a function of the task. In the present case, as well as in the Fleck study [4], subjects essentially performed a relatively low-level, entry-level object categorization task, namely target detection (‘*target present or not?*’). However, in the case of radiological images, the radiologist is typically required to also identify the object of interest, in addition to detecting it.

In the ultimate analysis, the significance of our study is not that it establishes that perceptual learning necessarily reduces SOS in all cases, but that it serves as proof of an important principle: That perceptual learning *can* reduce SOS.

## Figures and Tables

**Table 1:** The experimental conditions during SOS testing blocks in Experiment 1.

Condition Name	Description	Number of Trials per Block	Overall Prevalence
L target only (or ‘L-only’)	Image contained a single L target	10	~16.7%
H target only	Image contained a single H target	10	~16.7%
L and H targets (or ‘L-and-H’)	Image contained one L target and one H target (i.e., dual target image)	10	~16.7%
No target	Image contained no target	30	50%

**Table 2:** Experimental conditions during SOS testing blocks in Experiment 2.

Condition Name	Description	Number of Trials per Block	Overall Prevalence
L target only (or ‘L-only’)	Image contained a single L target	10	25%
H target only	Image contained a single H target	10	25%
L and H targets (or ‘L-and-H’)	Image contained one L target and one H target (i.e., dual target image)	10	25%
No target	Image contained no target	10	25%

## Data Availability

Data supporting the reported results can be obtained from the Corresponding Author (J.H.) upon reasonable request.

## Supplementary Materials

The following supporting information can be downloaded at: https://www.mdpi.com/article/10.3390/vision6030049/s1, Figure S1: Distribution of a specific type of ‘false positive’ responses during the SOS task before vs. after camouflage training in Experiment 1; Figure S2: Distribution of a specific type of ‘false positive’ responses during the SOS task before vs. after camouflage training in Experiment 2; Table S1: Hermite modeling of response counts in Experiment 1: Summary of results; Table S2: Hermite modeling of response counts in Experiment 2: Summary of results.
