# Supplementary material for: Satisfaction of Search Can Be Ameliorated by Perceptual Learning: A Proof-of-Principle Study
Source: Vision (Basel). 2022 Aug 10;6(3):49. doi: 10.3390/vision6030049 (PMC9396993; doi:10.3390/vision6030049)
Supplement: Supplementary file 1 [file vision-06-00049-s001.zip › vision-1442833-supplementary.pdf]

Supplementary Material

Supplementary Figure S1. Distribution of a specific type of 'false positive' responses during the SOS task before vs. after camouflage training in Experiment 1. See text for details.

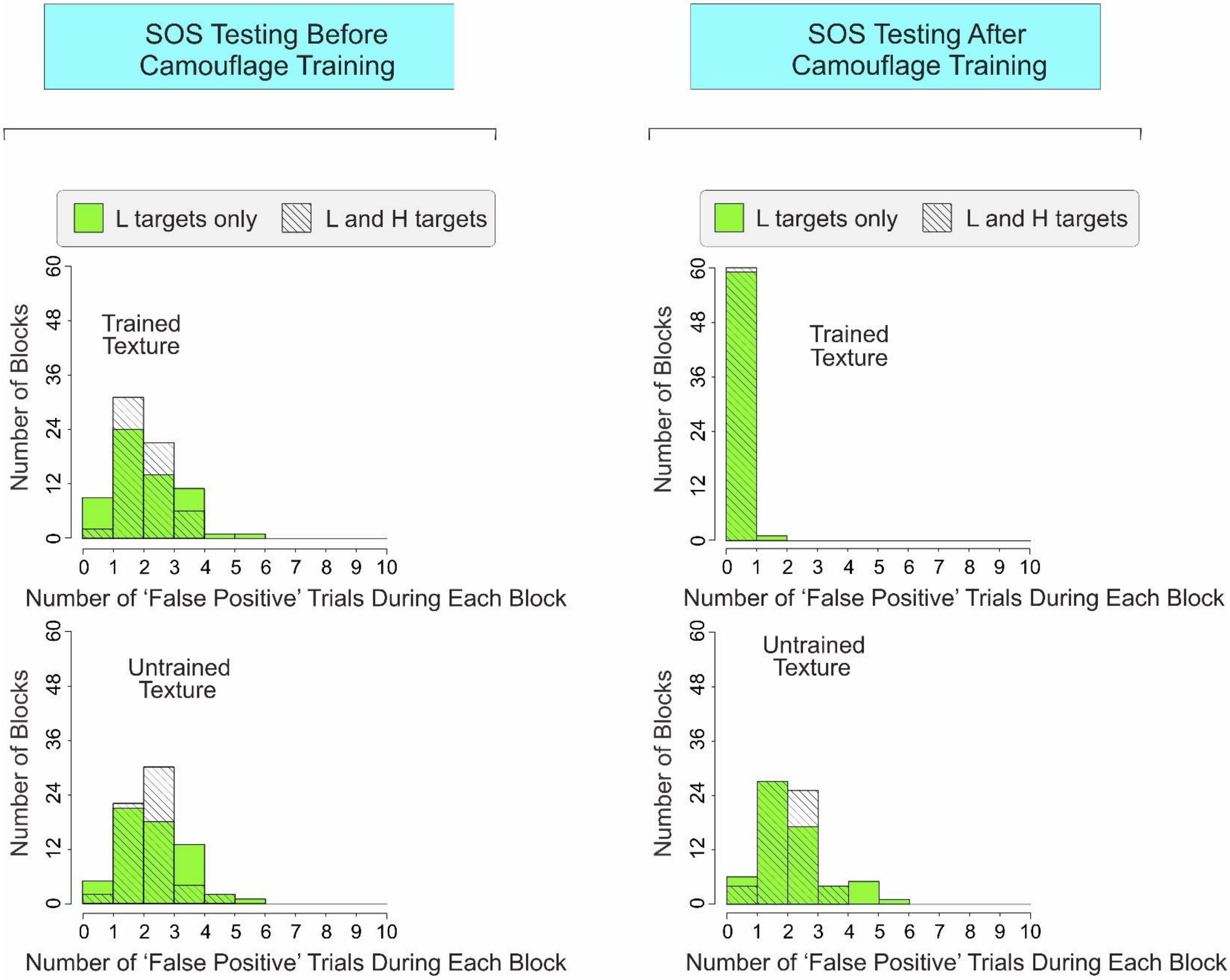

Supplementary Figure S2. Distribution of a specific type of 'false positive' responses during the SOS task before vs. after camouflage training in Experiment 2. See text for details.

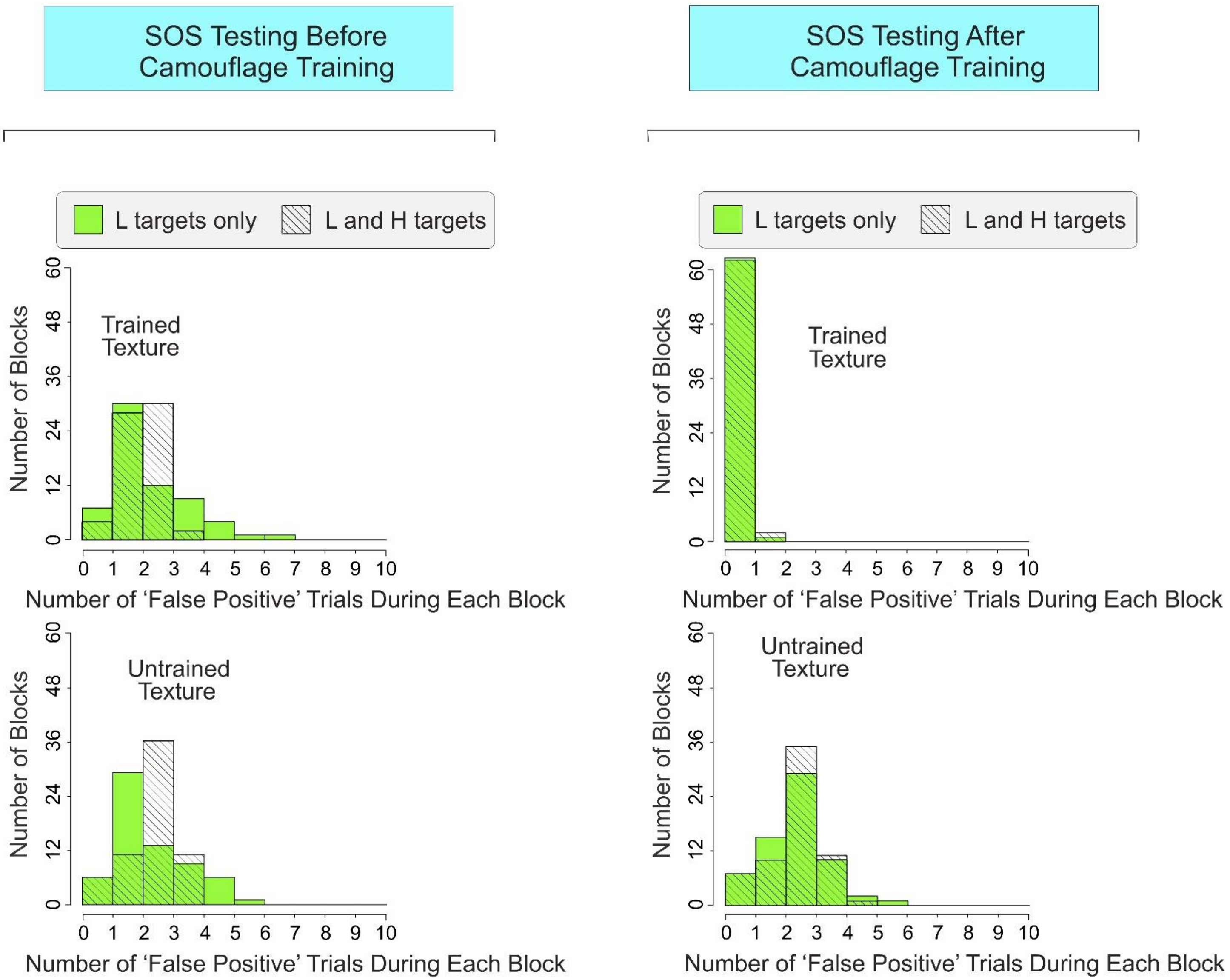

Supplementary Table S1. Hermite modeling of response counts in Experiment 1: Summary of results

Row #	**Independent Variable**	**Estimated Coefficient**	**Standard Error**	***z* value**	***p* value**
1	Null model (intercept only)	1.71	0.05	31.89	< 0.001
2	Testing condition (L-only *vs*. L-and-H)	0.14	0.03	5.69	< 0.001
3	Texture type (trained *vs*. untrained)	0.01	0.03	0.38	0.71
4	Training status (before *vs*. after)	0.20	0.03	6.27	< 0.001
5	Dispersion index	1.0	0.49	< -0.001	< 0.001

Supplementary Table S2. Hermite modeling of response counts in Experiment 2: Summary of results

Row #	**Independent Variable**	**Estimated Coefficient**	**Standard Error**	***z* value**	***p* value**
1	Null model (intercept only)	1.81	0.04	51.32	< 0.001
2	Testing condition (L-only *vs*. L-and-H)	0.09	0.02	3.69	< 0.001
3	Texture type (trained *vs*. untrained)	-0.008	0.02	-036	0.72
4	Training status (before *vs*. after)	0.15	0.02	5.97	< 0.001
5	Dispersion index	1.0	0.18	< -0.001	0.50
